# Establishing ground truth in the traumatic brain injury literature: if replication is the answer, then what are the questions?

**DOI:** 10.1093/braincomms/fcac322

**Published:** 2022-12-08

**Authors:** Diana R Priestley, Jason Staph, Sai D Koneru, Sarah M Rajtmajer, Andrew Cwiek, Samantha Vervoordt, Frank G Hillary

**Affiliations:** Department of Psychology, Penn State University, University Park, PA 16802, USA; Department of Psychology, Penn State University, University Park, PA 16802, USA; College of Information Sciences and Technology, Penn State University, University Park, PA 16802, USA; College of Information Sciences and Technology, Penn State University, University Park, PA 16802, USA; Department of Psychology, Penn State University, University Park, PA 16802, USA; Department of Psychology, Penn State University, University Park, PA 16802, USA; Department of Psychology, Penn State University, University Park, PA 16802, USA; Social Life and Engineering Sciences Imaging Center, Penn State University, University Park, PA 16802, USA

**Keywords:** traumatic brain injury, meta-science, replication crisis, reproducibility, citation network

## Abstract

The replication crisis poses important challenges to modern science. Central to this challenge is re-establishing ground truths or the most fundamental theories that serve as the bedrock to a scientific community. However, the goal to identify hypotheses with the greatest support is non-trivial given the unprecedented rate of scientific publishing. In this era of high-volume science, the goal of this study is to sample from one research community within clinical neuroscience (traumatic brain injury) and track major trends that have shaped this literature over the past 50 years. To do so, we first conduct a decade-wise (1980–2019) network analysis to examine the scientific communities that shape this literature. To establish the robustness of our findings, we utilized searches from separate search engines (Web of Science; Semantic Scholar). As a second goal, we sought to determine the most highly cited hypotheses influencing the literature in each decade. In a third goal, we then searched for any papers referring to ‘replication’ or efforts to reproduce findings within our >50 000 paper dataset. From this search, 550 papers were analysed to determine the frequency and nature of formal replication studies over time. Finally, to maximize transparency, we provide a detailed procedure for the creation and analysis of our dataset, including a discussion of each of our major decision points, to facilitate similar efforts in other areas of neuroscience. We found that the unparalleled rate of scientific publishing within the brain injury literature combined with the scarcity of clear hypotheses in individual publications is a challenge to both evaluating accepted findings and determining paths forward to accelerate science. Additionally, while the conversation about reproducibility has increased over the past decade, the rate of published replication studies continues to be a negligible proportion of the research. Meta-science and computational methods offer the critical opportunity to assess the state of the science and illuminate pathways forward, but ultimately there is structural change needed in the brain injury literature and perhaps others.

## Teaser

Priestley et al. report that the ever-expanding brain injury literature and scarcity of testable hypotheses hinder progress toward reliable science. When coupled with more rigorous approaches (pre-registration; strong, falsifiable hypotheses), meta-science and computational methods will improve our ability to track meaningful replication of the fittest hypotheses and transform the neurosciences.

## Graphical Abstract

**Graphical abstract:**
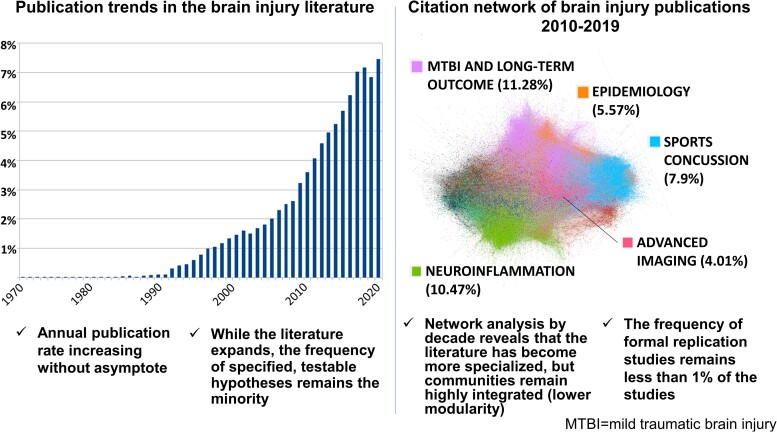

## Introduction

For over a decade, investigators have increasingly contended with concerns about reliability and reproducibility in science across a range of disciplines, including cancer research,^1^ economics^2^ and the clinical neurosciences.^3–6^ Emerging from these concerns are widespread efforts to increase the reproducibility of research and replicate published findings. To address the methodological and systemic barriers to performing quality replication studies, initiatives for open science,^7,8^ data and code sharing,^9,10^ and formalized pre-registration efforts^11^ are becoming increasingly commonplace. There have even been proposals for adjustments to the ways in which we move through the scientific process, wherein sequences of studies are conceptualized as opposed to isolated efforts, and investigators can adjust and re-evaluate the strength of hypotheses as data is accumulated.^12^

For many, the crisis has been an inspiration to revisit the most fundamental scientific principles: objectivity, healthy scepticism, and confirming hypotheses. Despite the groundswell of emphasis on increasing reliability, there remain important obstacles to achieving it. Nosek and colleagues have emphasized the need to evaluate the success of initiatives over the past decade designed to increase replicability, and this outcome remains unknown.^13^ Making matters worse, researchers are faced with the ever-expanding scientific literature,^14,15^ and what we refer to here as a ‘discovery-through-volume’ culture. In this period of high-volume science, one concern is the appearance of scientific advancement because of unmatched prolificacy. There are a number of problems with this rapid expansion of the scientific literature including the daunting task of determining which findings should be the focus for replication efforts.^16^ How, then, do we go about determining which hypotheses are worth pursuing further, and subsequently, which findings have sufficient evidence to be considered ‘ground truth’? Using a meta-science approach, we aim to track the primary goals within one neuroscience community, the study of traumatic brain injury (TBI), and how the community’s hypotheses are being framed and tested. The goal is to determine the primary themes that have evolved in the literature, the quality of the hypotheses being proposed, and the work being done to formally replicate hypotheses. The procedures and data from this meta-science approach are publicly available for application to a range of literatures within the clinical neurosciences (or any for that matter) with the goal of clarifying the primary themes within a literature, what has been established, and what work ideally should be replicated moving forward.

In order to assess the impact of individual papers within a body of literature, one common approach is to use a publicly available database to perform a citation analysis and identify those that are most cited. Web of Science (WoS), PubMed and Google Scholar are popular choices for this goal, as researchers can easily perform keyword searches, sort by the number of citations and then further characterize the most influential papers (defined as the highest citation). Citation counting as a means to track influential hypotheses has been used in the neurosciences to examine the TBI,^17–19^ neurorehabilitation,^20^ neurosurgery,^21^ and addiction^22^ literatures. Citation counting is quite useful for efficiently highlighting influential papers, researchers, journals, and institutions, identifying common research practices, and differentiating popular research topics from areas that require further investigation. This approach has limitations, however, when the goal is to identify areas where science would benefit from replication most. For example, the inclusion of review papers or critiques, which often comprise a large proportion of the most cited papers, obscures our ability to identify influential empirical studies.^23^ Additionally, a ‘snapshot’ search does not allow for insights into scientific trends over time. Finally, while citation count offers considerable evidence for the importance of a paper to a field, using it as the sole indicator of support for a hypothesis can be misleading. For example, Serra-Garcia *et al.*^24^ demonstrate that non-replicable papers are commonly cited more than papers whose findings were successfully reproduced. This kind of manual review provides valuable insights but is time-consuming and can only provide insight into a set of hypotheses or a fraction of known literature. We, therefore, endeavoured to conduct a more comprehensive analysis by sampling from a defined literature, modelling the scientific community over time and examining the research themes that emerge via the types of hypotheses proposed and the efforts to replicate findings.

### Goals for the present study

Given concerns about the rate of publishing in the clinical neurosciences and reliability of scientific findings, the goal of this study was to sample from one area within the broader clinical neurosciences (i.e. the TBI research community) and track the major themes that have comprised the literature over the past four to five decades. We conducted a decade-wise network analysis to determine the scientific communities that shape this literature. We also aimed to examine the strength of the leading hypotheses, as well as any attempts to examine hypotheses through formal replication. We utilized two distinct search engines in order to create a within-study robustness check of the result and to determine the role of common search engines on how we access the literature (WoS; Semantic Scholar, SS). Finally, to maximize transparency, we provide a detailed procedure for the creation and analysis of our data set, including a discussion of each of our major decision points, to facilitate similar work in the neurosciences more broadly.

**Hypotheses and Exploratory Goals** (pre-registered with the Center for Open Science; Registration https://doi.org/10.17605/OSF.IO/2BW3P)

First, consistent with recent trends in publication rates across scientific disciplines,^25^ we hypothesized that (i) the number of publications examining the consequences of TBI is increasing annually. Second, we hypothesized that (ii) the TBI literature is becoming more modular, forming increasingly segregated subcommunities. Related to this, and based upon the increasing interdisciplinary nature of the clinical sciences, we hypothesized that (iii) while modular and specialized, the communities within TBI will show greater integration with disciplines outside of TBI (e.g. links to Alzheimer’s disease). That is, the study of TBI is presently undergoing a shift from a focus on trauma to predictors of outcome and long-term consequences of additional factors, including how brain injury interacts with demography, health, and risk of abnormal ageing. Finally, we hypothesized that (iv) the frequency of replication studies within the TBI literature has increased over the last four decades.

In addition to these four pre-registered hypotheses, we included two exploratory goals. First **(Exploratory 1),** the most influential hypotheses in the TBI literature for each decade spanning 1970 to 2019 were examined to determine the strength and specificity of the hypotheses of each decade’s 50 most cited empirical papers. Second **(Exploratory 2),** we aimed to document how replication efforts are conducted within the field.

## Materials and methods

Our goal was to examine how the TBI literature has changed over time, document replication efforts, and understand the most influential (i.e. cited) hypotheses in this research community. To do so, we established a set of common search properties which could be applied across different datasets, search tools, and article metadata properties. We used a set of keywords and phrases that reliably identify articles in our focus area when applied to specific article record fields. When testing keywords, we evaluated sample results against a predetermined list of journals (Supplementary Table 1). Keywords were modified until papers from all expected journals were represented. The final set of keywords for our search was ‘(traumatic brain injury) OR (concussion) OR (closed head injury) OR (neurotrauma)’. Searches were performed, at a minimum, in the abstracts and titles, and were combined with the Boolean ‘OR’ operator. We did not limit our search to any specific range of publication dates, empirical paper types (e.g. primary analyses, meta-analyses, archival data analysis) or journals. Papers not published in English were excluded. In order to examine the robustness of our search outcome, we conducted separate searches in two widely used search engines.

### Web of Science search

We performed a *topic search* in the WoS Core Collection database on 18 June 2021 using our minimal standard search properties. The WoS ‘topic search’ queries author keywords and *Keywords Plus*, as well as article title and abstract. This initial search yielded 80 162 results. With the goal of focusing analyses on empirical work, we excluded publications that were not tagged by WoS as ‘articles’ (i.e. reviews, book chapter and conference summaries), after which 53 818 publications remained. We then sorted the results from highest to lowest based on citation count. We exported tab-delimited (Win, UTF-8) files, including the ‘full record and citing references,’ for all papers whose citation count was >10, which yielded 27 484 publications.

### Semantic Scholar search

There are algorithmic differences in how search engines conduct any given search. For this reason, we collected the second set of data from a separate citation database, SS, to verify the results of the network analysis. We aimed to follow a similar procedure to the above WoS export, noting that the design of SS is different from that of WoS.^26^ For example, unlike WoS, SS does not provide for Boolean searching, has limitations on filters that can be applied and does not allow users to export search results. For this reason, we were guaranteed some variation in the searches. Given these known differences, we aimed to determine the robustness of the network findings by comparing two distinct search outputs.

The complete SS corpus released on 1 June 2021 was downloaded for analysis in the form of JSON objects including 193 946 444 records. We conducted a keyword search with the minimal standard search properties listed above that queried titles and abstracts and included results from all years. This search yielded 122 090 records. Again, the SS corpus does not contain an exact field-to-field match per record with WoS; our searches inside the SS corpus therefore could not include fields unique to WoS such as Keywords Plus, Author Keywords, or the ‘Article’ tag. After eliminating papers with 10 or fewer citations, the final SS data set included 28 248 unique publications. Overall, the WoS search was more selective focusing on empirical work, while the SS search was more exhaustive.

#### Use of Web of Science and Semantic Scholar data sets

Important factors in our decision to use WoS and SS included ease of use, thorough indexing of journals and features such as the citation reporting tool. Networks were created using both WoS and SS data sets to examine the robustness of the results. However, for the goals to examine the types of hypotheses in the literature and attempts to replicate findings, we focused analysis on the WoS data set. This decision was based upon several of the WoS search capabilities but most importantly the desire to filter for influential empirical work when coding for the most cited hypotheses and identifying replication studies.

### Coding/analysis methods

#### Network analysis (hypotheses 2 and 3)

Decade-wise citation networks were generated for the WoS and SS data sets (1980–2019) with nodes representing unique papers and directed edges representing *cited* and *cited by* relationships using Neo4j.^27^ We extracted nodes and edges including both ‘inCitations’ and ‘outCitations’ using Python data processing. Citations were filtered such that all papers within the dataset effectively avoid single hops to papers not represented in the search (note: to examine within and without network citations, single hops not of interest were retained and quantified separately). Post-processing included extraction of citations between communities using Python scripts and filtering into subgraphs such that nodes and their inter-connections are isolated to the decade of interest. The Louvain algorithm for community detection was applied to undirected networks to identify communities within each decade. The labels were then combined with the edge information from the complete network to form edge relationships between papers in communities from different decades. Network findings were visualized using Gephi.^27^ For each detected community across all decades, one study team member with content expertise (FGH) examined the study title and goal of the top 10 most cited papers to assign community labels. For an exhaustive list of top papers and their corresponding labels, please see the shared data file titled: Community Labels in our GitHub repository.

#### Establishing the most influential hypotheses (Exploratory 1)

In order to track the most influential hypotheses, we sorted all records exported from WoS into their publication decade (1970s–2010s), which were then filtered by citation count (highest to lowest). We reviewed full-text PDFs of the most cited records to remove non-relevant publications (i.e. book chapters, consensus statements, literature reviews, non-TBI papers). Once we identified the 50 most cited *empirical* papers in the TBI field per decade (a total of 250 papers), we coded for hypothesis strength. We only considered hypotheses included in the introduction and/or methods sections in order to exclude ‘post hoc’ hypotheses. Papers that did not include explicit hypotheses were categorized as ‘exploratory’. For papers that did include hypotheses, each hypothesis was coded based on its quality, specifically: (i) some expectation, (ii) readily directional and falsifiable, (iii) directional, falsifiable *and* with an alternative hypothesis or an explicit statement of what sets of findings would *not* support the hypothesis. Coding was completed by two independent raters, and the degree of inter-rater reliability was assessed through the use of the weighted Cohen’s Kappa statistic. All disagreements in ratings were resolved by a third independent rater making a tie-breaking decision. Overall, there was a substantial agreement [*k* = 0.81, CI (0.78, 0.88), with 13% (*n* = 39)] of the total hypotheses requiring the additional reconciliation step.

#### Examining replication efforts (Hypothesis 4 and Exploratory 2)

It was also a goal to track the frequency of replication efforts. To determine the incidence of references to study replication, we conducted a second WoS search using our original terms, with the addition of the keyword ‘replica*’ in order to identify all papers that may have contributed to the discussion of reproducibility within the TBI literature {topic search = [(traumatic brain injury) OR (concussion) OR (closed head injury) OR (neurotrauma)] AND (replica*)}. Again, WoS permitted the selection of empirical work, so the resulting publications were filtered to those tagged as ‘articles.’ The full records of all papers in this final set, a total of 550, were then exported for further review. Of note, in order to be inclusive of all replication efforts, there was no filter for citation numbers.

Based upon this initial search, the abstracts for 550 papers were initially reviewed to exclude non-relevant publications including papers that (i) reference replication of clinical features of disease or injury; (ii) recommend future replication studies; (iii) reference DNA replication; (iv) are not written in English; and (v) do not investigate TBI. Based upon these exclusionary criteria, a team of reviewers selected a total of 125 papers for a full review, with each paper reviewed once. Full-text PDFs of these 125 papers were then manually coded by a single reviewer. In any case, where there was uncertainty, coding was resolved by a group discussion. Our primary goal was to identify ‘*classic replication (CR)*’ studies. For our purposes, a CR is an empirical study that tests either replicability (similar or identical methods in a different sample), robustness (identical data, different analysis) or reproducibility (similar or identical methods in an identical sample).^13^ To determine if a paper was a CR, we reviewed the abstract, introduction/goals, and methods sections of each paper. Key requirements of CRs were references to previous publications along with (i) discussion of the associated findings that were to be replicated and/or (ii) explicit evidence of an attempt to recreate the methodology (examples of acceptable language are included in Supplementary Table 2). Of the 125 papers, 40 met these criteria. A secondary goal was to determine the frequency of less formal replication work that serves as *corroborative evidence* (Ce) for a finding, irrespective of the effort for replication. Papers considered Ce were those that did not meet CR criteria but that still maintained focused discussion that linked the study results to a greater literature, with specific mention of replication or reproducibility in the introduction, procedures, or methods (examples provided in Supplementary Table 2). It is important to note that Ce language can be similar to that used in CR papers in some cases; however, the essential distinction is that Ce papers *lack* any discussion of recreating methodology. Following categorization as CR or Ce, all 125 papers were also coded according to the following criteria: (i) replication: same or similar method, different data; (ii) reproducibility: different lab, same data, same method; (iii) robustness: same data different analysis; (iv) other (e.g. replicating an idea using different data and non-identical approach); (v) non-standard, statistical replication; (vi) case study; (vii) any within-study reliability check/replication (same or diff method or data); and (viii) development or psychometric validation of survey/scale. Given these categories, the CRs (*n* = 40) were classified as 1, 2, 3, 6 or 8 and the CEs (85) were coded as 4, 5, 6, 7, or 8.

Furthermore, to examine replication attempts within and across laboratories, the nature of the study was recorded with respect to ‘within lab’ replication attempts (at least one author had to be listed on both the original and replication papers) or a ‘between lab’ attempt (no overlapping authors). To determine the reported success of the attempt, the *Results* and *Discussion* sections of each paper were reviewed for evidence presented by the authors. Papers were coded as: (0) failure to replicate (original findings were not supported); (1) successful replication (i.e. findings are in agreement with the original); (2) mixed evidence; (3) unclear.

#### Images

Figures 4 and 5 were produced in ggplot2.^28^ The graph inset seen in Fig. 5B was completed with image editing software. Network findings (Fig. 2 and 3) were visualized using Gephi.^29^

## Results

### Examining Hypothesis 1

The first goal of the study was to determine the trajectory of TBI publications over time. The primary hypothesis was that the number of publications in TBI is increasing without a clear asymptote.

**Figure 1:**
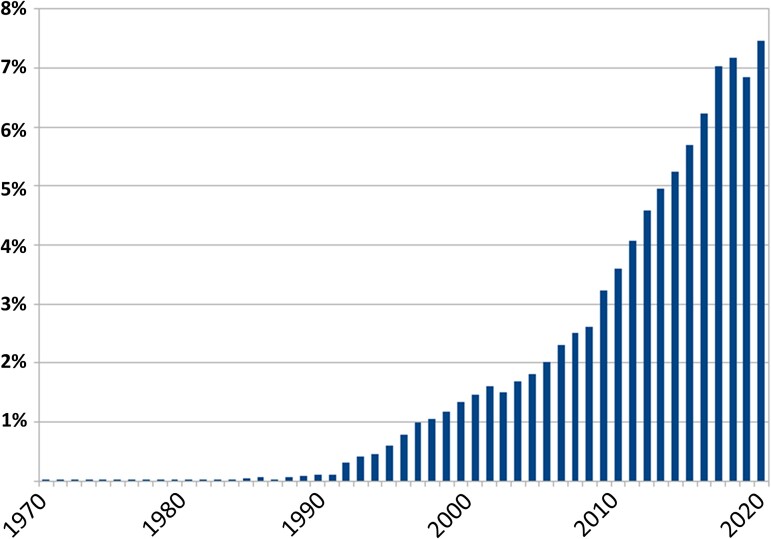
**Publication trends in the TBI literature.** The y-axis is the percentage of the total number of publications from 1900 to 2020 (*n* = 52 620) and the x-axis is the year based upon the WoS search. The data are graphed by binning the total in each year from 1970 to 2020 (of note, years prior to 1970 contributed little to the total and were removed to aid in visualizing change). N = 7 records were not included due to formatting irregularities. NOTE: there were no restrictions for citation count in order to be inclusive for most recent years.

Primary results indicate a clear increase in annual publication rate and much of this growth has occurred over the last decade. When considering papers published since 1900 (data prior to 1970 is available but not visualized in Fig. 1), more than 50% of the entire literature has been published in the last 10 years and 35% in the last five years.

### Examining hypotheses 2 and 3

**Figure 2:**
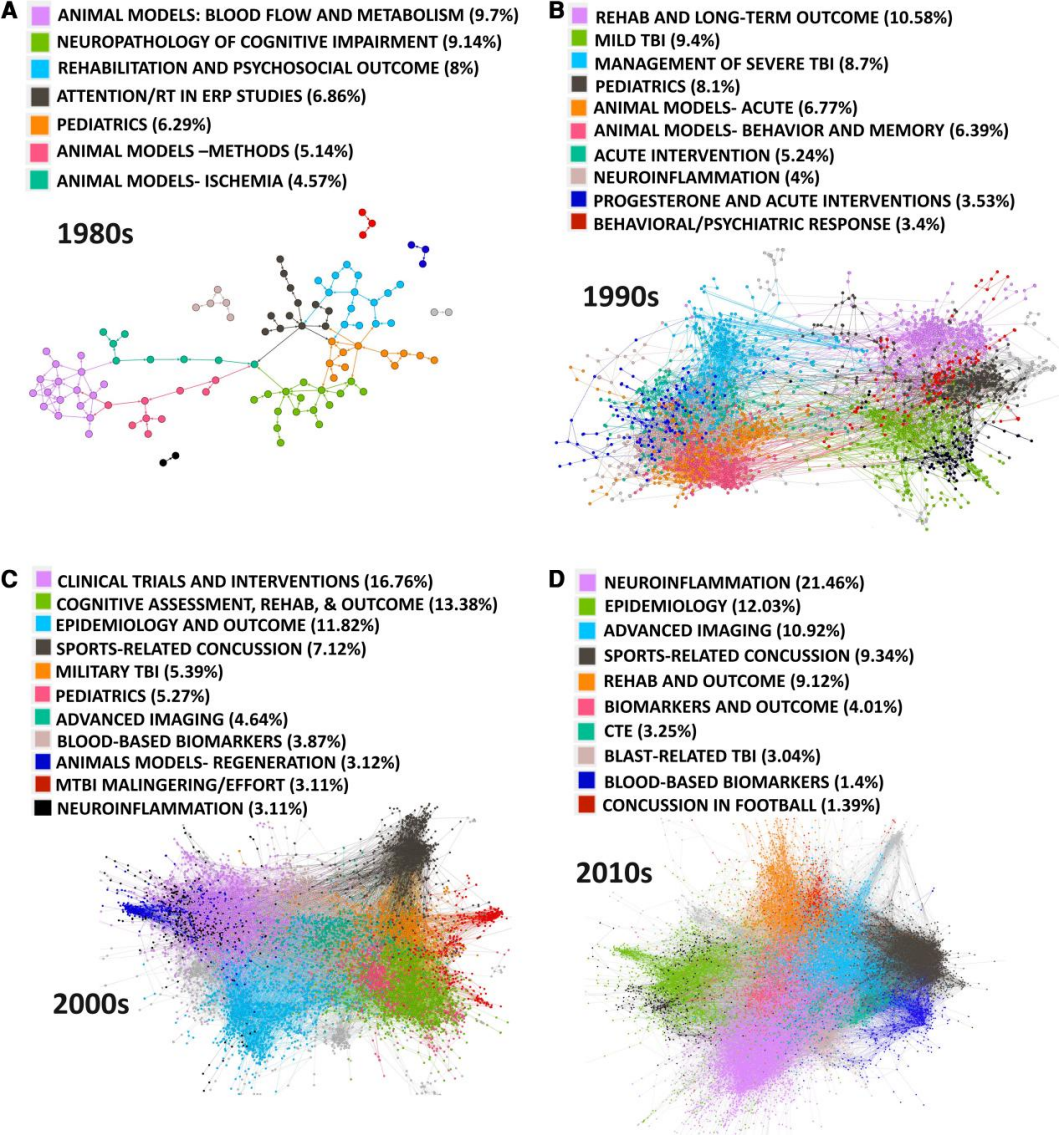
**WoS citation network.** Network results for the WoS search separated by decades 1980–2019, *n* = 27 193 total records with more than 10 citations, largest 7–10 communities are colour coded. The total number of nodes (edges) per decade: 1980s = 175 (127), 1990s = 3148 (10 481), 2000s = 9190 (41 763), and 2010s = 14 680 (77 763). RT = reaction time; ERP = event-related brain potential; CTE = chronic traumatic encephalopathy; mTBI = mild traumatic brain injury. NOTE: in order to visualize networks, some papers that were extreme outliers (represented as a great spatial distance from a module) are not depicted in the current renderings.

**Figure 3:**
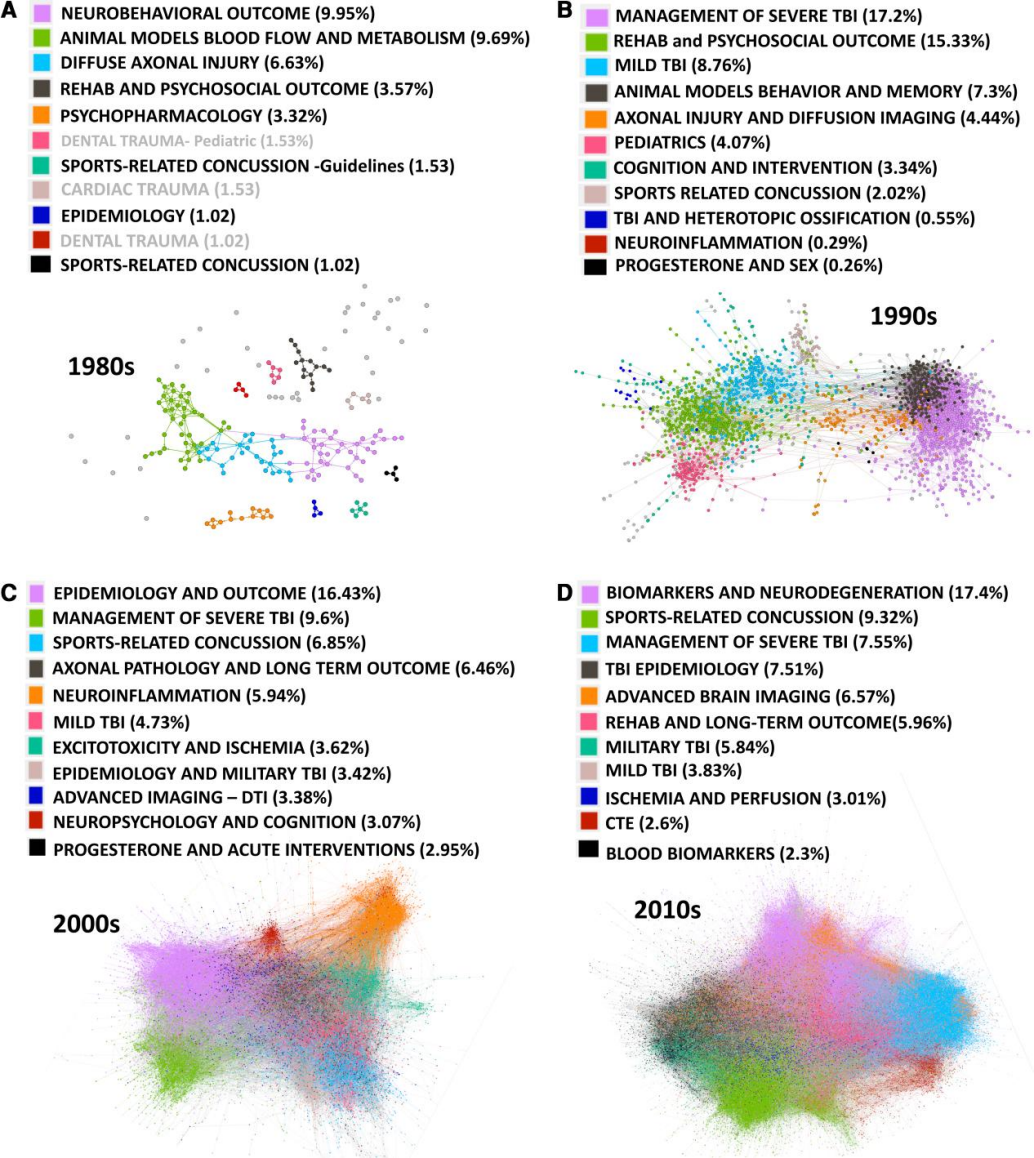
**SS citation network.** Network results for the SS search separated by decades 1980–2019, *n* = 28 248 total records with more than 10 citations, largest 7–10 communities are colour coded. The total number of nodes (edges) per decade: 1980s = 392 (268), 1990s = 2727 (7995), 2000s = 8959 (55 700), and 2010s = 16 170 (143 879). Abbreviations: CTE = chronic traumatic encephalopathy; TBI = traumatic brain injury; DTI = diffusion tensor imaging. NOTE: in order to visualize networks, some papers that were extreme outliers (represented as a great spatial distance from a module) are not depicted in the current renderings.

A second goal of the study was to conduct a citation network analysis decade by decade from the 1980s through 2019 with data from two citation databases. It was expected that more distinct communities have emerged within the TBI literature over time. We also expected to find that TBI work has become more integrated with other research communities over time; specifically, that the ratio of within-network citations to total citations has decreased from 1980 to 2019. Graph theoretical analysis of bibliographic networks reveals important evolution in the study of TBI over four decades. Findings demonstrate that network strength (total number of edges) has steadily increased from 1980 to 2019. This was present in both WoS and SS searches and is consistent with the overall publication growth. Network findings are presented in Fig. 2A–D and 3A–D.

### Changes in community modularity

It was hypothesized that greater specialization in the TBI research communities would emerge over time and this would be observed as increased network modularity. We also expected that while the scientific communities would become more specialized, TBI research would simultaneously become more integrated with other research communities (e.g. Alzheimer’s Disease literature) over time. We examined the interdisciplinary nature of each decade by testing the ratio of outside-network citations (bibliographic references to papers not within the original dataset) to total bibliographic citations. We anticipated that the ratio of outside-network citations to total citations has increased from 1980 to 2019.

Contrary to the primary hypothesis, the TBI research communities’ modularity peaked in the 1980s and became progressively less segregated over the following three decades [modularity Q scores: for WoS (1980s: 0.755, 1990s: 0.624, 2000s: 0.681, 2010s: 0.619); for SS (1980s: 0.752, 1990: 0.525, 2000s: 0.509, 2010s: 0.442)]. While the hypothesis that community segregation would increase along with increased specialization was not supported, there was some evidence that community specialization and strength (the number of nodes and connections) increased in the absence of increased community modularity. With regard to specialization, the number of identified communities grew from 7 to 10 between the 1980s and 1990s, resulting in the emergence of several communities (mTBI, concussion, blast-related head injury) where one previously existed (e.g. mTBI). Moreover, the size and strength of the top 5 communities increased each decade in both analyses. For example, looking at the 1980s in the WoS data set, the top five communities maintained relatively high community segregation (high modularity) while accounting for 40% of the total number of citations. By comparison, the total number of citations accounted for by the top five communities climbed to 44% in the 90 s, to 54% in the 2000s, and finally to 63% in the 2010s. Thus, the top five communities began to account for an increasing percentage of the literatures’ citation count in both searches (see Fig. 2A–D and 3A–D).

With regard to the second hypothesis that TBI research is becoming more interdisciplinary, the ratio of within/without citations was 4% in the 1980s, 10% in the 1990s, 13% in the 2000s and 16% in the 2010s, indicating that either the nodes outside the network are being adopted (i.e. included in a cluster) or that the TBI literature is becoming more self-referential and less integrative. Given that modularity decreases over time and the communities are less segregated, we interpret the within/without citation finding to indicate the latter—that the TBI literature is becoming more inclusive of other literatures and more interdisciplinary.

### Examining exploratory Analysis 1

In order to track emergent and influential themes in TBI research, we further aimed to examine the most highly cited hypotheses in each decade spanning 1970 through 2019.

**Figure 4:**
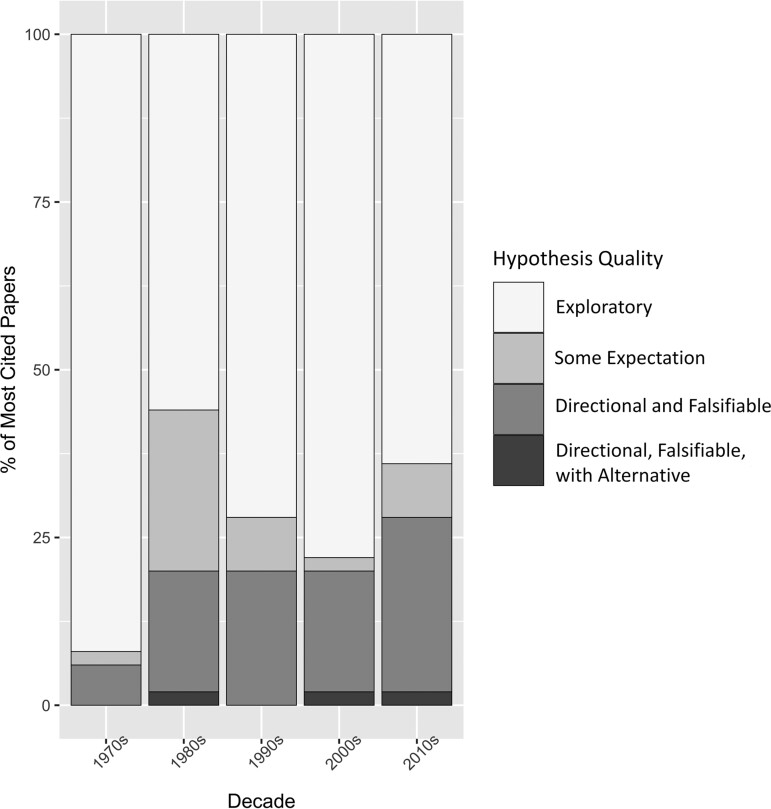
**Trends in the strength of TBI hypotheses by decade.** The y-axis is the percentage of the top 50 papers in each decade and the x-axis is the decade in which they were published based upon the WoS data set. The data are graphed by binning the top 50 most cited empirical TBI papers per decade from 1970 to 2019, for a total of *n* = 250 records. NOTE: papers that included more than one hypothesis were categorized based upon their *strongest* hypothesis so that they were not overrepresented in the graph.

As seen in Fig. 4, there was a general trend toward greater hypothesis specificity from the 1970s to the 2010s. The number of papers labelled as exploratory decreased over time, and there was also some increase in hypotheses that were testable with clear expectations about findings. Specifically, publications in the 1970s were almost exclusively exploratory in nature (92%), whereas 44% of publications in the 1980s included explicit hypotheses. The inclusion of alternative hypotheses in studies was rare; a total of 3 were provided within our entire set of 250 papers.

Results revealed that the number of highly cited papers requiring review to reach 50 empirical works increased over time, indicating that the literature may be moving from empirical work as a source of influence toward reviews and position papers. For example, in the 1970s and 1980s, 94% of the papers that required review were empirical works, as compared to 55 and 42% in the 2000s and 2010s, respectively.

### Examining Hypothesis 4 and exploratory Analysis 2

It was a goal to examine the frequency of replication attempts over time. We hypothesized that the number of CR studies being published is increasing over time, but that this number remains low when compared to the total number of publications. We also aimed to document how replication efforts are conducted within the field.

**Figure 5:**
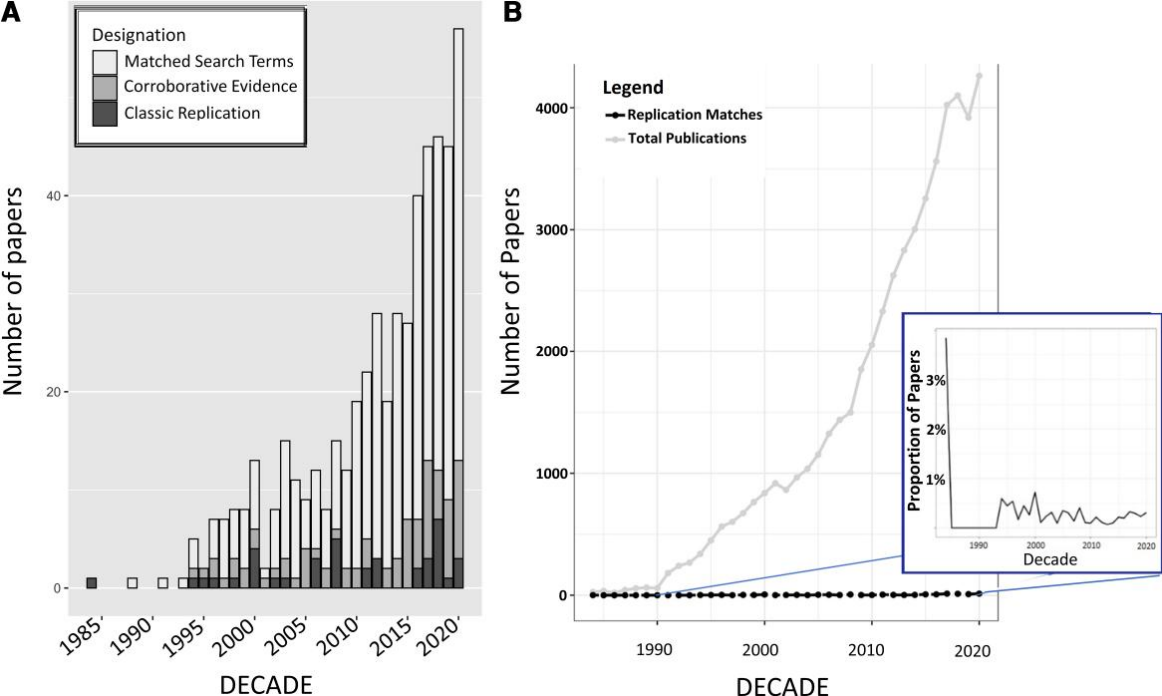
**Replication studies in the TBI literature.** (**A**) The y-axis is the number of publications and the x-axis is the year. Within the 57 210 papers in the TBI literature based upon the WoS search, 550 matched our search term (replica*). Further review revealed that the data set included 40 CRs and 85 CEs, with 425 remaining papers that matched the search terms but did not fit into either of these categories. The data are graphed by binning the total in each year from 1984 to 2020. (**B**) The y-axis is the number of publications and the x-axis is the year. In the inset, the y-axis is the percentage of the total number of publications that are replication matches and the x-axis is the year. NOTE: the box inset represents the proportion of studies matching the replication search to the total number of publications. The elevation in the 1980s is likely attributable to the sparse publication rate.

Results indicated that there has been an increase in references to replication more generally that mirrors the literature growth (Fig. 5A). However, after standardizing that growth against the total number of publications, the frequency of studies matching the search terms for replication remained relatively flat (see inset, Fig. 5B).

Upon review of the 550 abstracts matching the replication search, 396 non-relevant papers were excluded (e.g. those referencing animal models of TBI, recommending future replication studies, referencing DNA replication, not written in English, not specific to TBI, etc.). The 154 remaining studies, therefore, underwent a full PDF review. Of these, an additional 29 were excluded (*n* = 2 were non-English papers; *n* = 27 were not relevant to the search). The final 125 publications were then coded into categories. First, we determined that 32% (*n* = 40) were CR studies, while 68% (*n* = 85) were CEs. Given that our WoS dataset included 57 210 empirical papers in TBI, CRs and CEs make up approximately 0.07% and 0.15% of the TBI literature, respectively.

The 125 publications were also coded into categories, independent of their CR or Ce designation: (i) replication (same or similar method, different data), *n* = 30; (ii) reproducibility (different lab, same data, same method), *n* = 0; (iii) robustness (same data different analysis), *n* = 0; (iv) other (e.g. replicating idea using different data and non-identical approach), *n* = 52; (v) non-standard, statistical replication, *n* = 0; (vi) case study, *n* = 6; (vii) any within-study reliability check/replication (same or diff method data), *n* = 11; (viii) scale development/psychometric analysis, *n* = 26.

The reported success of these replication attempts is detailed in Table 2. Of note, CRs reported less success in reproducing results of original publications (62.5%) than CEs (77.6%). Additionally, within-laboratory replications reported more success than between-laboratory replications, both for CRs (76% versus 40%) and for CEs (90% versus 68.6%).

## Discussion

In this study, we assess the evolution of TBI research through a meta-science and network analysis approach. There were three related goals for this study. The first was to examine the overall throughput of TBI researchers with a focus on publication volume. Second, we conducted a network analysis of community structure to document the primary content areas that have been central to TBI work over the decades. The third was a focused analysis of efforts to replicate empirical TBI studies as well as the quality and types of hypotheses being posed over time. The outcome of each of these goals is summarized in-turn below.

### Science-by-volume approach to advance the neurosciences

The data from our WoS review indicate that there has been recent and unprecedented growth in the size of the brain injury literature, with the steepest growth in publication rates occurring between 2008–2018 (Fig. 1). Based on papers matching our search terms, since 2016, the TBI literature has been growing by over 4000 papers annually, representing an increase of 5–7% relative to the total number of empirical papers published since 1900. This growth in publication rate does not appear to be isolated to the study of TBI. A supplemental WoS analysis to examine the neurosciences broadly with the search terms ‘(neuro*) OR (behaviour) OR (brain) OR (cerebr*)’ revealed a similar publication trajectory (see Supplementary Figure 1A and B). The current publication throughput in the neurosciences dwarfs the early windows of this search (1970s and 1980s).

These data are also observable in the SS analysis. One caveat to this finding is that the WoS review, which mirrors the SS review in growth, more systematically selects empirical work. The dramatic increase in the publication trajectory in TBI, therefore, does not appear to be a function of a disproportionate proliferation of reviews, critiques, or scientific community summaries (e.g. position papers). Given the lack of tools to manage such a large annual scientific output, researchers face monumental challenges in verifying the veracity of claims and determining the most fruitful hypotheses to be tested moving forward.

Many have voiced concern about the immense volume of science, notably Nobel Laureate in physics Peter Higgs who lamented in the popular press that if he were to pursue an academic job today, he would not be hired because of concerns about his ‘productivity.’^30^ In response to this, there have been targeted criticism of publish-or-perish mandates^31^ and programmatic efforts internationally to make science more deliberate through de-emphasis of publication rates in academic circles.^32^ However, one could easily see that the argument that ‘less science is better’ is, at face value, difficult to justify. Others might even doubt the real cost to high-volume science—the ‘how can more data, more investigators, more papers be bad?’ perspective (see^15^). However, there are legitimate considerations for how a culture of science-by-volume is problematic for the *reliability* of science, in particular when research being conducted is not actively testing and eliminating viable hypotheses.^16^

Ultimately, efforts to ‘slow science’ may be counterproductive because they fail to acknowledge the advancements in data aggregation and analysis afforded by high-performance computing and rapid scientific communication through technology. For instance, researchers have recently begun to employ data-driven approaches including machine learning (ML) techniques and other forms of artificial intelligence (AI) to distill and assemble the literature.^33,34^ Other examiners have made use of prediction markets^35–37^ which have been shown to be highly accurate in predicting replicability, although they remain quite costly and time-consuming to run. Both approaches show enormous potential for computational approaches and Big data to advance reproducibility efforts.

Still, a critical question to be answered is whether the proliferation of published findings in the neurosciences has been met with unparalleled scientific advancement. The repeated failure of acute clinical trials in TBI and other areas of the clinical neurosciences offers little reassurance that progress is matching our scientific pace.^38–40^ Let us accept the argument for a moment that our rate of publishing has outstripped scientific advancement.^12,14,41^ The next clear question is what should be done about this, given known incentive structures. It would be difficult to condemn any academic hoping to meet the publication arc in their discipline when facing professional questions about training, hiring, and promotion.^31,42^ Even so, there remains a very clear need to determine how this unprecedented volume of published work has translated to advancements in the understanding of TBI pathophysiology and the improvement of outcomes in patients.

## Community analysis

### Evolution of the scientific communities examining brain injury

The primary hypothesis that the study of brain injury would evolve from a relatively uniform community with a general focus on brain injury toward more specialized communities was not supported by increased modularity as expected. In fact, the opposite was observed and network modularity was highest in the 1980s, declined in the 1990s, and then became more stable after the 2000s. This was true for both the WoS and SS reviews, which again were different searches that emphasized empirical work and a more comprehensive search including reviews and critiques, respectively. While network modularity did not increase over time, the number of distinct communities did grow with increasing specialization by the 2010s. Those communities also became increasingly robust, accounting for a larger overall percentage of citations each year. For example, in the 1980s, the top 5 communities were highly segregated (high modularity) but accounted for only 40% of the total number of citations. During the 1990s and 2000s, the communities became increasingly interconnected and stronger; the total number of citations accounted for by the top 5 communities climbed to 44% in the 1990s, to 54% in the 2000s, and then to 63% in the 2010s. These data are mirrored in the SS review although the overall percentages are lower, likely because that search did not filter for empirical articles, which may dilute this effect. Of note, a supplementary analysis of the SS data (see Supplementary Figure 2), in which articles cited 10 times or less were *not* removed, revealed similar results.

It, therefore, appears that the TBI literature over the past 3–4 decades has become more specialized while remaining highly integrated (observable as relatively stable modularity). As one example of specialization, in the 1990s WoS data, mTBI/concussion was a single identifiable cluster capturing a range of mild injury phenomena. However, by the 2010s, four separate mTBI communities emerged including sports concussion/mTBI, blast-related TBI, chronic traumatic encephalopathy (CTE), and a very small cluster focused on concussion in American football (1.9% of the overall citations). As another example, the CTE literature emerged in the 2000s and matured into a distinct community in the 2010s. So, while the hypothesis that these communities would become more distinct was not supported because all emerging communities remained connected to each other, there does appear to be greater specialization in the study of TBI emerging over the past three decades. This finding parallels previous work suggesting that research communities become more integrated over time,^43,44^ and that division of labour is a natural result of interdisciplinary work.^45^ The implications of greater specialization in science have been discussed in many fields (microbiology,^46^ mathematics,^47^ sociology^48^). Despite some disadvantages, it has been argued that increased specialization is both a natural evolution of science and a necessity—uncovering new evidence in such sizable literature requires greater concentration and focus.^46^ Given this, we suggest that the specificity of our hypotheses must match the specialization of our research; doing so will permit the identification of the most profitable pathways forward and facilitate transdisciplinary collaboration.

### Research methods as drivers of communities

Changing community structure over four decades also offers indicators that research methods may play an important role in advancing research themes. In the 1980s there was a very clear separation between animal and human models of TBI. This trend continued in the 1990s, where some links exist between the literatures (acute intervention work and management of severe TBI), but for the most part, the communities relied upon distinct methods and questions (see Fig. 2 and 3, *panels A* and *B*). This trend begins to change in the 2000s (evident in both WoS and SS reviews) with the communities of advanced imaging and blood-based biomarkers serving as connectors between animal and human models of TBI (Fig. 2 and 3, *panel C*). In the SS data set, this community separation is bridged during the 1990s with the examination of axonal injury using diffusion tensor imaging. As of the 2000s, animal and human work were highly integrated with the topic of inflammation as well as critical reviews/summary statements unifying multiple literatures. By the 2010s, while animal and human work remain critical to the study of TBI, these literatures are fully integrated across separate research streams.

If permitted to speculate about the role of methods in shaping this literature, what appears evident is that the influence of methods has changed over time. For example, the development of distinct TBI animal models dominant in the 1980s (e.g. weight drop, fluid percussion) were vehicles for discovery. This is also evident in the methods for the acute management of severe TBI in humans (e.g. elevated intracranial pressure, acute ischaemia assessment, and hyperglycolysis; see Fig. 2, *panels A* and *B*). These distinct communities disappeared by the 2000s, perhaps because the methods are accepted and infused within larger research agendas as opposed to being the scientific focus of any specific research community. By the 1990s, other literatures appeared as conduits between human and animal work including advanced imaging (Fig. 2 and 3, *panel C*) and blood-based biomarkers (Fig. 2, *panel C*).

It remains to be determined, however, if the TBI sciences have moved from the developmental/exploratory phase to a phase of deeper conceptual problem-solving. On one hand, there has been a dramatic growth in specialized research communities; based upon our data, however, this occurs in the context of no greater specificity in the nature of the questions being asked. There is, of course, a critical need for exploratory work in all scientific disciplines. In fact, Scheel and colleagues make the interesting point that exploratory work is vital during periods where links between theory and measures to be tested are not well established. Under these conditions, highly specific hypotheses may be uninformative.^49^ Instead, there should be a greater focus on establishing sound relationships between method and theory, as opposed to the universal emphasis on statistical significance testing seen today. In this way, scientific exploration establishes the groundwork for deeper conceptual questions.

The natural tension grows, however, when widespread exploration occurs in an environment of high-volume science. In the absence of rigorous theory building, unchecked exploration may simply circle back to what is believed to be known (excessive citation of known work, or the ‘canon’) resulting in a slowing of creative and novel ideas.^14^ The modern TBI literature is now at least four decades mature with high publication rates in large established subcommunities (see Fig. 2D and 3D), and yet there is still a clear need to determine the field’s scientific ‘anchors,’ identify critical areas requiring additional support, and dedicate resources to focused efforts for replication.

### Tracking influential hypotheses

Analyses of the 50 most highly cited empirical papers per decade revealed that many of the most influential papers in the TBI literature have been exploratory in nature. The vast majority of the hypotheses for all decades were categorized as maintaining ‘some expectation’, with little indication as to the direction or magnitude of the studied effect (see Table 1). While publications in the 2020s may bear different results, there appeared to be no obvious change in the quality of proposed hypotheses between 1980 and 2020 (see Fig. 4). Hypotheses that were either exploratory or maintained ‘some expectation’ constituted more than 70% of the literature from 1980 to 2020. During that same time, hypotheses that could be definitively tested occurred 20–25% of the time and the strongest hypotheses (directional, falsifiable, with an alternative provided) remained rare (3–7%). Unfortunately, the challenge with relying on exploration and general predictions has a long history in the behavioural sciences representing important challenges to falsifiability and theory building.^50,51^ Understandably, the emergence of novel methods is commonly accompanied by tentative hypotheses (for brain imaging in TBI see^52^ and more generally see,^53,54^) but even with the growth and maturity of the scientific communities examined here, we find little associated change in the strength of the hypotheses.

**Table 1:** TBI hypothesis quality by decade

	70s	80s	90s	00s	10s
Empirical, TBI-related works (*n* = 50 per decade)					
Papers reviewed to reach 50 empirical, TBI-related works	53	53	70	90	119
Exploratory papers	46 (92%)	28 (56%)	36 (72%)	39 (78%)	32 (64%)
Papers including explicit hypotheses	4 (8%)	22 (44%)	14 (28%)	11 (22%)	18 (36%)
Total number of explicit hypotheses per decade					
	*n* = 9	*n* = 34	*n* = 28	*n* = 15	*n* = 29
Some expectation	1 (11%)	19 (56%)	11 (39%)	3 (20%)	12 (41.5%)
Directional and falsifiable	8 (88%)	14 (41%)	17 (61%)	11 (73%)	16 (55%)
Directional, falsifiable, with alternative	0 (0%)	1 (3%)	0 (0%)	1 (7%)	1 (3.5%)

The most cited papers in each decade (1970s–2010s) from the WoS export were reviewed to identify the top 50 empirical publications in each, for a total of 250 papers. Book chapters, consensus statements, literature reviews, and non-TBI papers were excluded. Hypotheses were then extracted from the full-text PDFs and characterized as *exploratory* or *explicit*. Explicit hypotheses were then further coded in terms of specificity: (i) some expectation; (ii) directional and falsifiable; or (iii) directional, falsifiable, and with an alternative hypothesis provided. NOTE: some papers provided more than one explicit hypothesis.

**Table 2:** Replication studies by type and success

	Classic replications (CR) (*n* = 40)	Replication efforts (RE) (*n* = 85)
Successful	25 (62.5%)	66 (77.6%)
“Failed”	9 (22.5%)	6 (7.1%)
Conflicting evidence	6 (15%)	9 (10.6%)
Interpretation unclear	0 (0%)	4 (4.7%)
Between study	40 (100%)	70 (82.4%)
Within study	0 (0%)	15 (17.5%)
Between lab*	15 (37.5%)	35 (41.2%)
Within lab**	25 (62.5%)	30 (35.3%)
Unclear	0 (0%)	6 (7.1%)
N/A	0 (0%)	14 (16.5%)
	Between Lab CR* (*n* = 15)	Between Lab RE* (*n* = 35)
Successful	6 (40%)	24 (68.6%)
“Failed”	8 (53.3%)	4 (11.4%)
Conflicting evidence	1 (6.7%)	4 (11.4%)
Interpretation unclear	0 (0%)	3 (8.6%)
	Within Lab CR** (*n* = 25)	Within Lab RE** (*n* = 30)
Successful	19 (76%)	27 (90%)
“Failed”	1 (4%)	0 (0%)
Conflicting evidence	5 (20%)	3 (10%)
Interpretation unclear	0 (0%)	0 (0%)

Note: two sets of results for the CR and Ce categories.

The frequency of published TBI work without well-formulated hypotheses is concerning, but when matched with unparalleled growth in the publication rate, it creates a perfect storm for challenges to scientific reproducibility. Interestingly, the number of papers reviewed per decade in order to reach 50 of the most cited empirical papers did increase with each decade and this effect was evident even when filtering for non-empirical work in the WoS search. We interpret this effect as the scientific community's effort to manage the volume of science by relying, increasingly, on *expert* panel position papers, reviews, and critiques. For example, in the 1970s and 1980s, 94% of the top papers reviewed were empirical works, as compared to 55 and 42% in the 2000s and 2010s, respectively (Table 1). One advantage to this transition is that, commonly, the authors of position papers hold critical experience and vision for the field. While this may be adaptive to managing the deluge of scientific publishing, a primary consequence is that it distills scientific input to interpretation by a select scientific community—an important consideration given that half or more of the most cited works in the most recent decades are non-empirical—and this is using a search that aims to identify empirical reports. The most highly cited papers in the SS were largely critical reviews or position papers.

### Trends in replication efforts

At least part of what the current study documents is the challenge in identifying formal efforts to replicate specific hypotheses. While there are searchable resources for researchers to examine and quantify paper retractions (e.g. Retraction Watch), there is no similar resource available to investigators to synthesize the outcome of replication attempts. Because a range of methods to test the reliability of findings may occur in the absence of a formal replication attempt (or even the term *replication*), it was our goal to assess the frequency of explicit attempts to replicate as well as the use of the language of replication. While imperfect, we tracked the frequency of the use of this term in the field to serve as a barometer of change and discussion in the field—if not an absolute marker for the true frequency of replication attempts in the TBI sciences. While we anticipated that this would result in only a fraction of studies, the total number of studies fulfilling the criteria was surprisingly low (*n* = 550) and the percentage of formal replication attempts compared to the annual publication rate remains flat over the four decades studied (see Fig. 5B).

When comparing CRs and CEs in terms of success, authors of CR studies reported more often (22.5%) that their attempt ‘failed’ (i.e. that their findings did not match those of the original paper) as compared to CEs that did not match design or sample (7.1%). Furthermore, failure to replicate was reported less when the effort was conducted within the same lab as the original work, both for CRs (4% failure within laboratories versus 53.3% between laboratories) and for CEs (0% failure within laboratories versus 11.4% between laboratories). This finding might be attributed to scientific reasons (original authors of a study maintain consistent procedures/methods), as well as human factors (e.g, implicit bias).

While the ideal frequency for study replication is unclear, if replication is to have an impact on the TBI literature, one could argue that these efforts should account for greater than <1% of the research agenda. The ∼50% failure rate for between-laboratory replications observed in these data speaks to the critical need for verification of all findings. If replication is to become a legitimate endeavour within the clinical neurosciences, it requires volitional effort and structures in place to support replication efforts. While the current review cannot be definitive, it demonstrates without much doubt that there remains a need for organized replication efforts supported within the clinical neuroscience community—efforts to conduct similar work are already supported in other scientific circles (see recent work in Cancer).^55,56^ The goal to formalize replication in any field is a non-trivial effort, requiring scientific experts working in concert to devise methods that permit reasonable conclusions regarding what constitutes a ‘replication’ (see ‘precommitment’ from Nosek and Errington, 2020).^57^ In the absence of a known structure and methodological standard for replication in the clinical neurosciences, efforts will continue to be rare events, represent idiosyncratic testing of hypotheses, and likely provide little convincing evidence that can advance the science. Without improving this structure, examiners do not have the tools to accurately answer questions regarding what is definitively known and, given the answer to this first question, what is the most important theory or hypothesis to test.

### Limitations and conclusion

There are several important caveats to the current findings and our interpretation. The most important is that citation networks represent but one approach to understanding the evolution of science. The quality of citations and citation topology (supporting/refuting) is vital in order to understand the impact of highly cited papers and the direction of science. SCITE is an excellent example of emerging technologies that permit coarse-grained analysis of citation typology through the use of machine learning to analyse the text surrounding a citation where it appears in the full text of the paper and, based on this surrounding text, classifies a citation as supporting, contrasting or neither. This is an active current literature in natural language processing attempting to better extract and assess research claims and supporting evidence. This work has been propelled by the release by AllenAI of SciFact, a benchmark data set of claims and supporting evidence (REF: https://aclanthology.org/2020.emnlp-main.609/) to support algorithm development and testing. At the present time, the MultiVerS software (REF: https://aclanthology.org/2022.findings-naacl.6/) performs best on this task (an active leaderboard is maintained here: https://leaderboard.allenai.org/scifact). From a different perspective, coarse analyses of citation type is also supported by Semantic Scholar; in that case, citations are classified as more or less ‘influential’ based on the section of the paper in which they appear, e.g. Background versus Methods (REF: https://www.nature.com/articles/547032a).

Adding this context beyond the simple presence of a citation is an important step forward and precisely the type of emerging technology that exemplifies opportunities for the research community to push past the current metrics and representations underlying bibliometrics and metanalyses. We note that these technologies are still very much in development, with, for example, the accuracy of the MultiVerS software currently sitting around 0.7 SCITE and MultiVerS both rely on the full text of the papers, and SCITE is proprietary. Once these tools are thoroughly vetted, the community—we believe—will have the opportunity and the responsibility to reimagine citation counts, citation networks, and subsequent analyses like the ones we have undertaken in this paper.

Separately, the manual coding of hundreds of papers with respect to study goals and hypotheses requires reliance on the evidence presented by the authors regarding support for *a priori* hypotheses and replication success. We did not gain access to the original data nor did we conduct verifying analyses. Separately in our examination of the most influential papers, we defined ‘influence’ by citation count. While citation count is an objective standard and provides a quantity that is readily analysed, this is a heuristic approach that binarizes citation relationships treating them with identical importance. Also, while the goal of including WoS and SS was to gauge the robustness of the findings (i.e. identical search terms, different search engines, and filters), the two searches are not identical due to the nature of the search algorithms. The outcome is that the graphs show some community differences which are at least partially driven by the significantly greater number of critical reviews in the SS search compared to the WoS search. Even so, the primary findings outlined in the Discussion remain robust to the specifics of each search (e.g. separation between animal and human work in the 1990s). Overall, it does appear that the two searches provide evidence for consistency in the changes in this literature over time. Finally, the replication analysis (Fig. 5A and B) was conducted using specific search terms and then manual coding of several hundred papers. It is possible that some researchers have published CRs and CEs but have not described them in those terms, causing us to omit relevant papers from our data set. While our search is unlikely to be exhaustive, we anticipate that the outcome of our search is not systematically biased with regard to how replication work has been conducted or its relative success.

In summary, the unparalleled rate of scientific publishing within the TBI literature combined with the scarcity of clear hypotheses in individual publications makes evaluating accepted findings and determining paths forward a daunting task. Additionally, while the conversation about reproducibility has increased over the past decade, the rate of published replication studies continues to be a negligible proportion of the research. Meta-science and computational methods offer the critical opportunity to assess the state of the science and illuminate pathways forward, but ultimately there is structural change needed in the TBI literature (and perhaps the clinical neurosciences broadly). Changes in the scientific culture should take the form of formalized support for researchers engaging in replication studies, rewarding these efforts such as through funding and career advancement, and developing structures to allow researchers to systematically search the outcome of replication efforts. In a scientific environment of finite resources available to examine a rapidly expanding literature, and a growing emphasis on replication, answering the question ‘What sets of findings are the most important to replicate?’ appears to be a crucial priority now more than ever.

## Supplementary Material

fcac322_Supplementary_Data

## Data Availability

All data analysed for this manuscript have been made publicly available (see https://github.com/PennStateBrainLab/replication) including a readme.txt file to facilitate use. We also share all code and scripts used for analysis. For any questions regarding access to the data, please contact the senior author at fhillary@psu.edu. NOTE: Certain data included herein are derived from Clarivate Web of Science. © Copyright Clarivate 2022. All rights reserved. NOTE: Semantic Scholar data source: Semantic Scholar API https://partner.semanticscholar.org/?utm_source=api.

## Abbreviations

Ce =: corroborative evidence
CR =: classic replication
MTBI =: mild traumatic brain injury
SS =: semantic scholar
TBI =: traumatic brain injury
WoS =: web of science

## References

[1] Nosek BA, Errington TM. Making sense of replications. Elife. 2017;6. 10.7554/eLife.23383 PMC5245957 28100398

[2] Camerer C, Dreber A, Forsell E, et al Evaluating replicability of laboratory experiments in economics. Science. 2016;351(6280):1433–1436. 26940865 10.1126/science.aaf0918

[3] Gelman A, Geurts HM. The statistical crisis in science: How is it relevant to clinical neuropsychology? Clin Neuropsychol. 2017;31(6–7):1000–1014. 28075223 10.1080/13854046.2016.1277557

[4] Botvinik-Nezer R, Holzmeister F, Camerer CF, et al Variability in the analysis of a single neuroimaging dataset by many teams. Nature. 2020;582(7810):84–88. 32483374 10.1038/s41586-020-2314-9 PMC7771346

[5] Kellmeyer P . Ethical and legal implications of the methodological crisis in neuroimaging. Camb Q Healthc Ethics. 2017;26(4):530–554. 28937337 10.1017/S096318011700007X

[6] Hillary FG, Medaglia JD. What the replication crisis means for intervention science. Int J Psychophysiol. 2020;154:3–5. 31082406 10.1016/j.ijpsycho.2019.05.006 PMC6842660

[7] Allen C, Mehler DMA. Open science challenges, benefits and tips in early career and beyond. PLoS Biol. 2019;17(5):e3000246. 31042704 10.1371/journal.pbio.3000246 PMC6513108

[8] McKiernan EC, Bourne PE, Brown CT, et al How open science helps researchers succeed. Elife. 2016;5. 10.7554/eLife.16800 PMC4973366 27387362

[9] Ferguson AR, Nielson JL, Cragin MH, et al Big data from small data: Data-sharing in the ‘long tail’ of neuroscience. Nat Neurosci. 2014;17(11):1442–1447. 25349910 10.1038/nn.3838 PMC4728080

[10] Martone ME, Garcia-Castro A, VandenBos GR. Data sharing in psychology. Am Psychol. 2018;73(2):111–125. 29481105 10.1037/amp0000242 PMC5920518

[11] Nosek BA, Ebersole CR, DeHaven AC, et al The preregistration revolution. Proc Natl Acad Sci U S A. 2018;115(11):2600–2606. 29531091 10.1073/pnas.1708274114 PMC5856500

[12] Nichols JD, Oli MK, Kendall WL, et al Opinion: A better approach for dealing with reproducibility and replicability in science. Proc Natl Acad Sci U S A. 2021;118(7):e2100769118. 10.1073/pnas.2100769118 PMC7896342 33568535

[13] Nosek BA, Hardwicke TE, Moshontz H, et al Replicability, robustness, and reproducibility in psychological science. Annu Rev Psychol. 2022;73:719–748. 34665669 10.1146/annurev-psych-020821-114157

[14] Chu JSG, Evans JA. Slowed canonical progress in large fields of science. Proc Natl Acad Sci U S A. 2021;118(41):e2021636118. 10.1073/pnas.2021636118 PMC8522281 34607941

[15] Shiffrin RM, Borner K, Stigler SM. Scientific progress despite irreproducibility: A seeming paradox. Proc Natl Acad Sci U S A. 2018;115(11):2632–2639. 29531095 10.1073/pnas.1711786114 PMC5856513

[16] Rajtmajer SM, Errington TM, Hillary FG. How failure to falsify in high-volume science contributes to the replication crisis. eLife. 2022;8(11):e78830. 10.7554/eLife.78830 PMC9398444 35939392

[17] Li L, Ma X, Pandey S, et al The most-cited works in severe traumatic brain injury: A bibliometric analysis of the 100 most-cited articles. World Neurosurg. 2018;113:e82-e87. 29409928 10.1016/j.wneu.2018.01.164

[18] Sharma B, Lawrence DW. Top-cited articles in traumatic brain injury. Front Hum Neurosci. 2014;8:879. 25414657 10.3389/fnhum.2014.00879 PMC4220681

[19] Karydakis P, Giakoumettis D, Themistocleous M. The 100 most cited papers about pediatric traumatic brain injury: A bibliometric analysis. Ir J Med Sci. 2020;189(1):315–325. 31418153 10.1007/s11845-019-02085-6

[20] Kreutzer JS, Agyemang AA, Weedon D, et al The top 100 cited neurorehabilitation papers. NeuroRehabilitation. 2017;40(2):163–174. 28222551 10.3233/NRE-161415

[21] Lipsman N, Lozano AM. Measuring impact in stereotactic and functional neurosurgery: An analysis of the top 100 most highly cited works and the citation classics in the field. Stereotact Funct Neurosurg. 2012;90(3):201–209. 22677634 10.1159/000337170

[22] Valderrama Zurian JC, Bueno Canigral FJ, Castello Cogollos L, et al The most 100 cited papers in addiction research on cannabis, heroin, cocaine and psychostimulants. A bibliometric cross-sectional analysis. Drug Alcohol Depend. 2021;221:108616. 33636599 10.1016/j.drugalcdep.2021.108616

[23] Zhang Y, Xiong Y, Cai Y, et al The 100 top-cited studies on neuropsychology: A bibliometric analysis. Front Psychol. 2020;11:550716. 33329180 10.3389/fpsyg.2020.550716 PMC7734023

[24] Serra-Garcia M, Gneezy U. Nonreplicable publications are cited more than replicable ones. Sci Adv. 2021;7(21):eabd1705. 34020944 10.1126/sciadv.abd1705 PMC8139580

[25] White K . Publications Output: U.S. Trends and International Comparisons: Executive Summary. Available: https://ncses.nsf.gov/pubs/nsb20206/executive-summary. 2022.

[26] Fricke S . Semantic scholar. J Med Libr Assoc. 2018;106(1).

[27] Neo4J. 2022.

[28] Wickham H . Ggplot2: Elegant graphics for data analysis. Springer; 2016.

[29] Bastian M, Heymann S, Jacomy M. Gephi: An Open Source Software for Exploring and Manipulating Networks. Third International AAAI Conference on Weblogs and Social Media 2009.

[30] Schekman R . How journals like Nature, Cell and Science are damaging science Available: https://www.theguardian.com/commentisfree/2013/dec/09/how-journals-nature-science-cell-damage-science. 2022.

[31] Kiai A . To protect credibility in science, banish “publish or perish”. Nat Hum Behav. 2019;3(10):1017–1018. 31602039 10.1038/s41562-019-0741-0

[32] Dijstelbloem H, Miedema F, Huisman F, et al Why science does not work as it should and what to do about it. Science in Transition. 2013.

[33] Altmejd A, Dreber A, Forsell E, et al Predicting the replicability of social science lab experiments. PLoS One. 2019;14(12):e0225826. 31805105 10.1371/journal.pone.0225826 PMC6894796

[34] Yang Y, Youyou W, Uzzi B. Estimating the deep replicability of scientific findings using human and artificial intelligence. Proc Natl Acad Sci U S A. 2020;117(20):10762–10768. 32366645 10.1073/pnas.1909046117 PMC7245108

[35] Forsell E, Viganola D, Pfeiffer T, et al Predicting replication outcomes in the many labs 2 study. J Econ Psychol. 2019;75:102117.

[36] Dreber A, Pfeiffer T, Almenberg J, et al Using prediction markets to estimate the reproducibility of scientific research. Proc Natl Acad Sci U S A. 2015;112(50):15343–15347. 26553988 10.1073/pnas.1516179112 PMC4687569

[37] Gordon M, Viganola D, Dreber A, et al Predicting replicability—Analysis of survey and prediction market data from large-scale forecasting projects. PLoS One. 2021;16(4):e0248780. 33852589 10.1371/journal.pone.0248780 PMC8046229

[38] Schwamm LH . Progesterone for traumatic brain injury — Resisting the sirens’ song. N Engl J Med. 2014;371(26):2522–2523. 25493975 10.1056/NEJMe1412951

[39] Stein DG . Embracing failure: What the phase III progesterone studies can teach about TBI clinical trials. Brain Inj. 2015;29(11):1259–1272. 26274493 10.3109/02699052.2015.1065344 PMC4667711

[40] Loannidis JPA, Greenland S, Hlatky MA, et al Increasing value and reducing waste in research design, conduct, and analysis. Lancet. 2014;383(9912):166–175. 24411645 10.1016/S0140-6736(13)62227-8 PMC4697939

[41] Bowen A, Casadevall A. Increasing disparities between resource inputs and outcomes, as measured by certain health deliverables, in biomedical research. Proc Natl Acad Sci U S A. 2015;112(36):11335–11340. 26283360 10.1073/pnas.1504955112 PMC4568675

[42] Nosek BA, Spies JR, Motyl M. Scientific utopia: II. Restructuring incentives and practices to promote truth over publishability. Perspect Psychol Sci. 2012;7(6):615–631. 26168121 10.1177/1745691612459058 PMC10540222

[43] Varga A . Shorter distances between papers over time are due to more cross-field references and increased citation rate to higher-impact papers. Proc Natl Acad Sci U S A. 2019;116(44):22094–22099. 31611374 10.1073/pnas.1905819116 PMC6825301

[44] Casadevall A, Fang FC. Field science—The nature and utility of scientific fields. mBio. 2015;6(5):e01259–01215. 26350968 10.1128/mBio.01259-15 PMC4600110

[45] Haeussler C, Sauermann H. Division of labor in collaborative knowledge production: The role of team size and interdisciplinarity. Res Policy. 2020;49(6):103987.

[46] Casadevall A, Fang FC. Specialized science. Infect Immun. 2014;82(4):1355–1360. 24421049 10.1128/IAI.01530-13 PMC3993417

[47] Morris R . Increasing specialization: Why we need to make mathematics more accessible. Social Epistemology. 2021;35(1):37–47.

[48] Leahey E, Reikowsky RC. Research specialization and collaboration patterns in sociology. Soc Stud Sci. 2008;38(3):425–440.

[49] Scheel A, Tiokhin L, Isager P, et al Why hypothesis testers should spend less time testing hypotheses. Perspectives on Psychological Science. 2021;16(4):744–755. 33326363 10.1177/1745691620966795 PMC8273364

[50] Meehl P . Theory-Testing in psychology and physics: A methodological paradox. Philos Sci. 1967;34(2):103–115.

[51] Morey RD, Lakens D. Why most of psychology is statistically unfalsifiable. 2016.

[52] Hillary FG . Neuroimaging of working memory dysfunction and the dilemma with brain reorganization hypotheses. J Int Neuropsychol Soc. 2008;14(4):526–534. 18577281 10.1017/S1355617708080788

[53] Poldrack RA, Farah MJ. Progress and challenges in probing the human brain. Nature. 2015;526(7573):371–379. 26469048 10.1038/nature15692

[54] Paret C, Unverhau N, Feingold F, et al Survey on open science practices in functional neuroimaging. Neuroimage. 2022;257:119306. 35595201 10.1016/j.neuroimage.2022.119306

[55] Errington TM, Denis A, Perfito N, et al Challenges for assessing replicability in preclinical cancer biology. Elife. 2021;10:e67995. 34874008 10.7554/eLife.67995 PMC8651289

[56] Errington TM, Mathur M, Soderberg CK, et al Investigating the replicability of preclinical cancer biology. Elife. 2021;10:e71601. 34874005 10.7554/eLife.71601 PMC8651293

[57] Nosek BA, Errington TM. Argue about what a replication means before you do it. Nature. 2020;583:518–520. 32694846 10.1038/d41586-020-02142-6

