# Supplementary material for: Establishing ground truth in the traumatic brain injury literature: if replication is the answer, then what are the questions?
Source: Brain Commun. 2022 Dec 8;5(1):fcac322. doi: 10.1093/braincomms/fcac322 (PMC9806718; doi:10.1093/braincomms/fcac322)
Supplement: fcac322_Supplementary_Data [file fcac322_supplementary_data.pdf]

Supplementary Table 1: Required TBI journals

Journal of Neurotrauma
Brain Injury
Brain Research
The Journal of Trauma and Acute Care Surgery
PLOS One
Journal of Neurosurgery
Journal of Head Trauma Rehabilitation
Archives of Physical Medicine and Rehabilitation
Experimental Neurology
Neuropsychology
Frontiers in Neurology
Frontiers in Systems Neuroscience
Behavioural Brain Research
NeuroImage
Brain Connectivity

Supplementary Table 2: Language included in Classic Replication (CR) Papers versus Corroborative Evidence (CE) papers

Classic Replication Language	Corroborative Evidence Language
“The methodology used was nearly identical to a prior study on…”[1]	"This finding replicates previous demonstrations of resting-state hyperconnectivity in TBI"[2]
"The present study followed a similar procedure except that a name-matching task was used."[3]	"The findings of the current study were largely consistent with previous research."[4]
"…the present study attempts to replicate and extend the procedures likely to be used by practitioners from the initial Millis et al. (1995) research."[5]	"We replicated the HIT method and confirmed several previous findings at the standard level of injury severity."[6]
"…replication of these data was attempted after detailed consultation (via email and phone) with the original authors” … “a research technician... traveled to the [original author’s laboratory] to learn their surgical approach and injury technique"[7]	"The secondary purpose was to replicate previously reported sex differences in total concussion symptoms, and performance on neurocognitive and balance testing."[8]
"To this end this research recreated the essential components of the Tsushima et al. investigation of repeated non- concussive head blows with youth athletes."[9]	"The present study was conducted to investigate whether the patterns of findings observed in adults with TBI would replicate in a pediatric sample."[10]

Supplementary Figure 1a: Annual neuroscience publication rate (all publications)

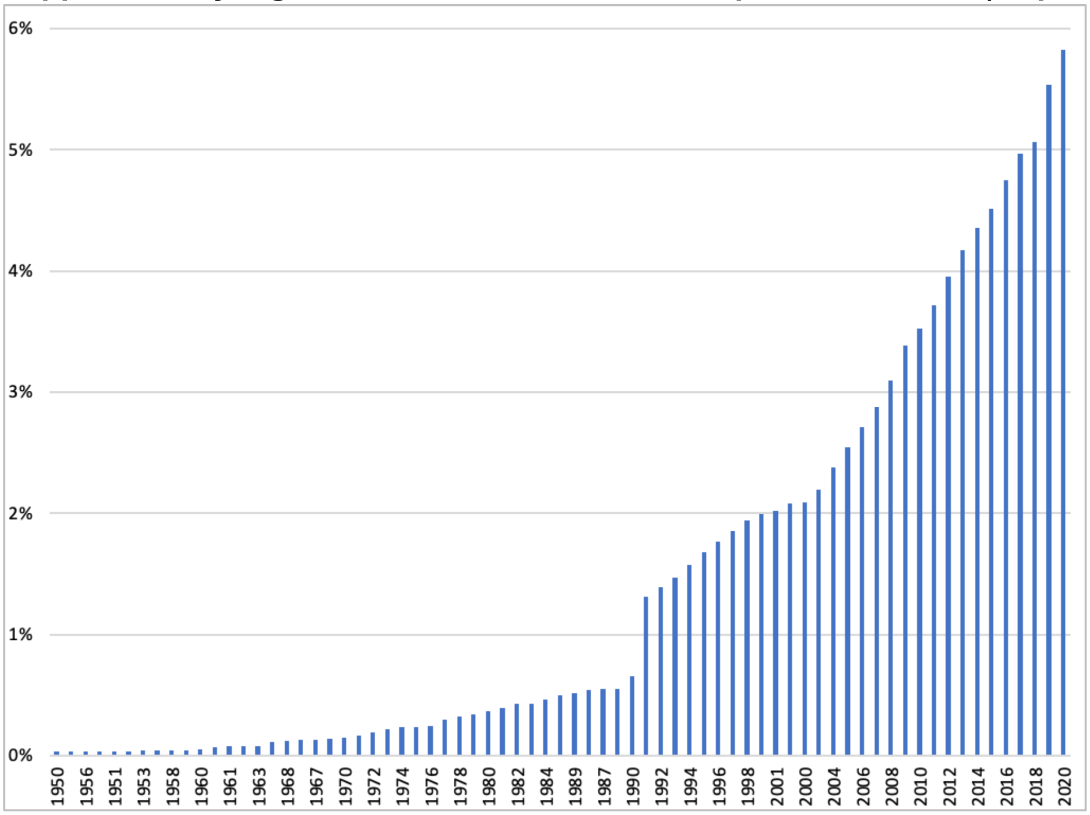

Supplementary Figure 1b: Annual neuroscience publication rate (articles only)

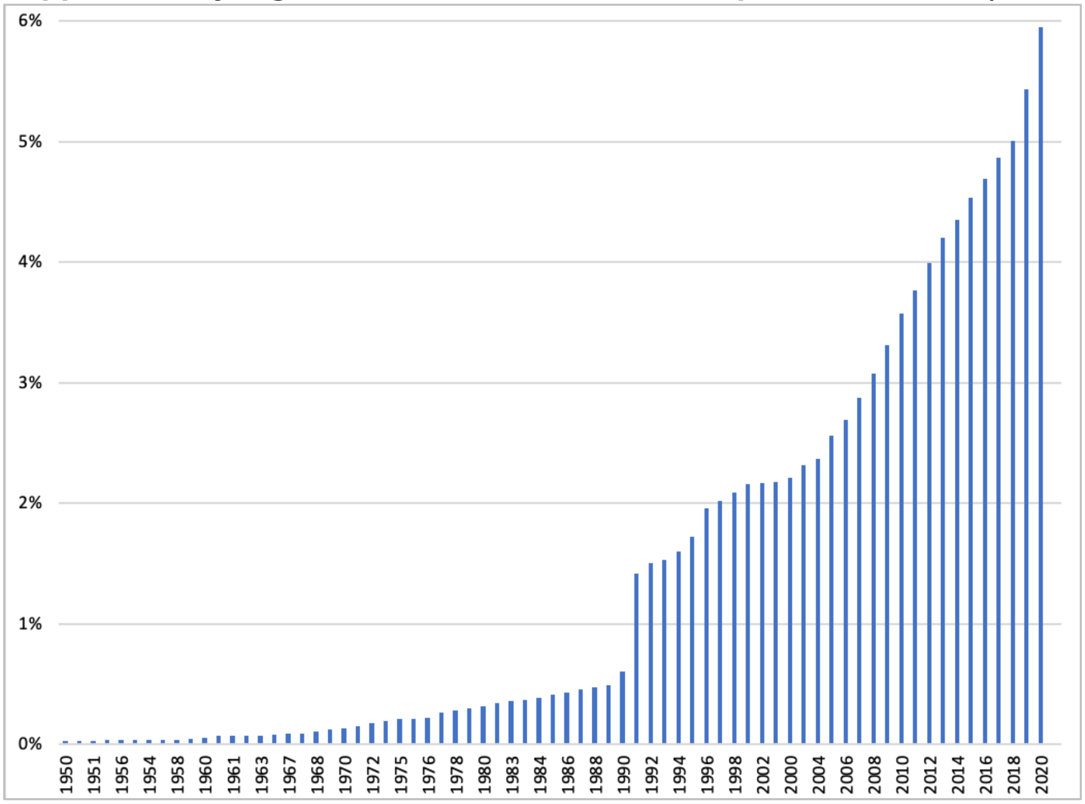

Supplementary Figure 2: Citation Network of publications in the TBI literature from the unfiltered Semantic Scholar (SS) search

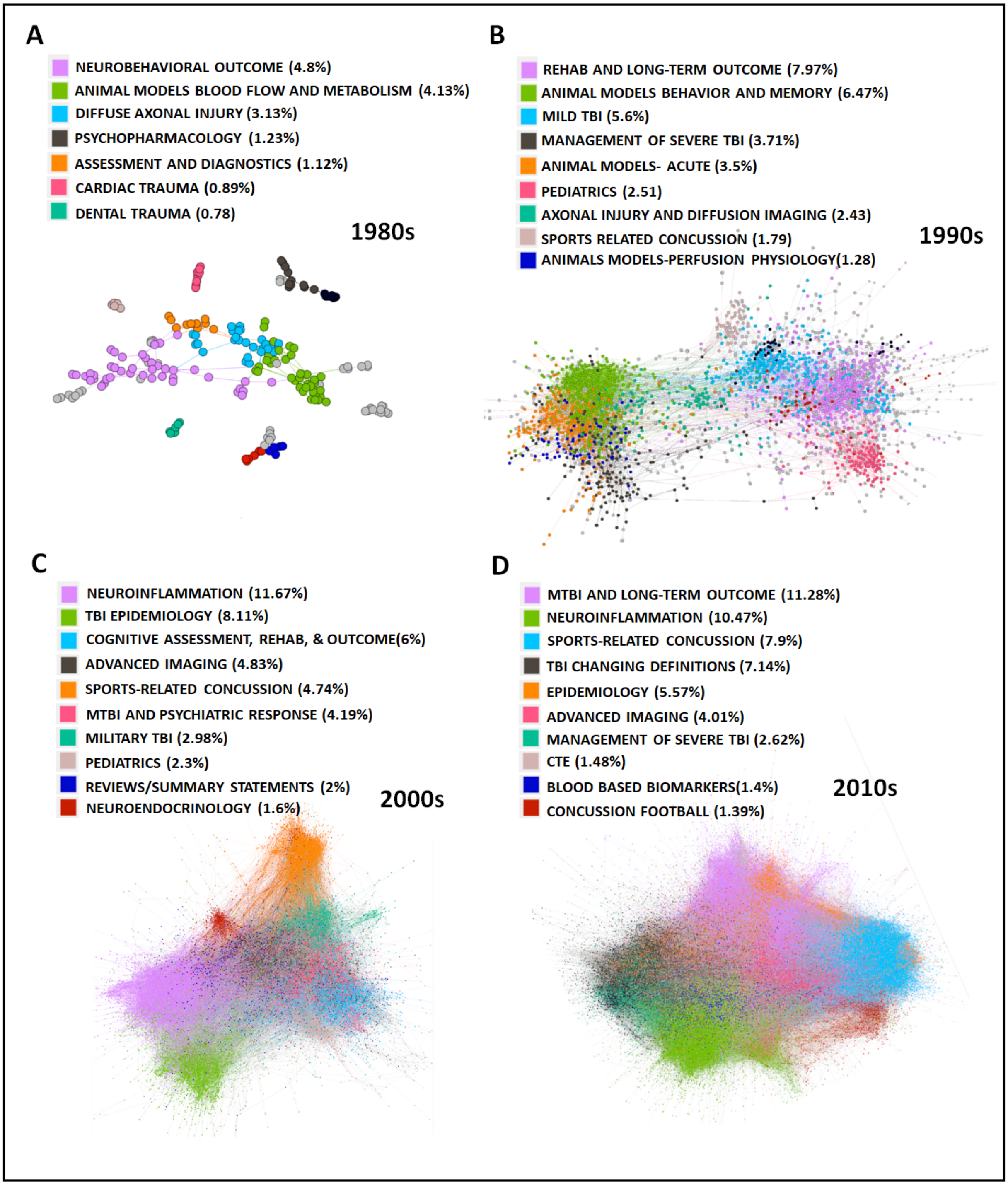

SEMANTIC SCHOLAR
A free, AI-powered research tool for scientific literature

LEGENDS

Supplementary Figure 1

Data were collected from Web of Science using the search terms ("neuro* OR brain OR cognition OR cerebr*"). The data were graphed by limiting results to publications between 1950 to 2020, and binning the total in each year. There were no restrictions for citation count in order to be inclusive for most recent years. **A)** All publications were included. The y-axis is the percentage of the total number of publications (n=3,413,540) and the x-axis is the year. **B)** Records were limited to those labeled with the WoS "article" tag. The y-axis is the percentage of the total number of "articles" within the search (n=2,448,352) and the x-axis is the year.

Supplementary Figure 2

Network results for the unfiltered SS search (including papers cited 10 times or fewer) separated by decades 1980-2019, n=76,470 total records, largest 7-10 communities are color coded. The total number of nodes (edges) per decade: 1980s=895 (360), 1990s=4855 (9,809), 2000s=17,727 (72,318), and 2010s=52993 (315,625). Abbreviations: CTE = chronic traumatic encephalopathy; mTBI = mild traumatic brain injury. **NOTE**: in order to visualize networks, some papers that were extreme outliers (represented as a great spatial distance from a module) are not depicted in the current renderings.

REFERENCES

1. Whyte J, Vaccaro M, Grieb-Neff P, et al. The effects of bromocriptine on attention deficits after traumatic brain injury: a placebo-controlled pilot study. Am J Phys Med Rehabil. 2008;87(2):85-99.
2. Hogeveen J, Aragon DF, Rogge-Obando K, et al. Ventromedial Prefrontal-Anterior Cingulate Hyperconnectivity and Resilience to Apathy in Traumatic Brain Injury. J Neurotrauma. 2021;38(16):2264-2274.
3. David H. K. Shum, Ken McFarland, Bain JD. Effects of Closed-Head Injury on Attentional Processes: Generality of Sternberg's Additive Factor Method. Journal of Clinical and Experimental Neuropsychology. 1994;16(4):547-555.
4. Corrigan JD, Horn SD, Barrett RS, et al. Effects of Patient Preinjury and Injury Characteristics on Acute Rehabilitation Outcomes for Traumatic Brain Injury. Arch Phys Med Rehabil. 2015;96(8 Suppl):S209-221 e206.
5. Sweet JJ, Wolfe P, Elizabeth Sattlberger, et al. Further Investigation of Traumatic Brain Injury Versus Insufficient Effort with the California Verbal Learning Test. Archives of Clinical Neuropsychology. 2000;15(2):105-113.
6. Putnam LJ, Willes AM, Kalata BE, et al. Expansion of a fly TBI model to four levels of injury severity reveals synergistic effects of repetitive injury for moderate injury conditions. Fly (Austin). 2019;13(1-4):1-11.
7. Popovich PG, Lemeshow S, Gensel JC, et al. Independent evaluation of the effects of glibenclamide on reducing progressive hemorrhagic necrosis after cervical spinal cord injury. Exp Neurol. 2012;233(2):615-622.
8. Sufrinko AM, Mucha A, Covassin T, et al. Sex Differences in Vestibular/Ocular and Neurocognitive Outcomes After Sport-Related Concussion. Clin J Sport Med. 2017;27(2):133-138.
9. Tsushima WT, Siu AM, Yamashita N, et al. Comparison of neuropsychological test scores of high school athletes in high and low contact sports: A replication study. Appl Neuropsychol Child. 2018;7(1):14-20.
10. Erdodi LA, Lichtenstein JD, Rai JK, et al. Embedded validity indicators in Conners' CPT-II: Do adult cutoffs work the same way in children? Appl Neuropsychol Child. 2017;6(4):355-363.
